# Off-Label Use of Ataluren in Four Non-ambulatory Patients With Duchenne Muscular Dystrophy: Effects on Cardiac and Pulmonary Function and Muscle Strength

**DOI:** 10.3389/fped.2018.00316

**Published:** 2018-10-23

**Authors:** Daniel Ebrahimi-Fakhari, Ulrich Dillmann, Marina Flotats-Bastardas, Martin Poryo, Hashim Abdul-Khaliq, Mohammed Ghiath Shamdeen, Bernhard Mischo, Michael Zemlin, Sascha Meyer

**Affiliations:** ^1^Department of Pediatric Neurology, Saarland University Medical Center, Homburg, Germany; ^2^Department of Neurology, Saarland University Medical Center, Homburg, Germany; ^3^Department of Pediatric Cardiology, Saarland University Medical Center, Homburg, Germany; ^4^Department of Pediatrics, Marienkrankenhaus St. Josef Kohlhof, Neunkirchen, Germany

**Keywords:** Translarna, Duchenne muscular dystrophy, DMD, non-ambulatory, nmDMD, stop codon read-through therapy, pulmonary function test, treatment

## Abstract

About 15% of Duchenne muscular dystrophy (DMD) cases are caused by point mutations leading to premature stop codons and disrupted synthesis of the dystrophin protein. Stop codon read-through therapy is available with the drug Ataluren (Translarna® by PTC Therapeutics). Following positive results in ambulatory nmDMD (non-sense mutation Duchenne muscular dystrophy) patients, Ataluren received conditional approval in ambulant nmDMD patients by the EMA in 2014. However, there are limited data on non-ambulatory nmDMD patients treated with Ataluren. Here, we report our experience in four non-ambulatory DMD patients. Routine investigations included cardiac function, pulmonary function tests and muscle strength. We compared changes in left ventricular fractional shorting, forced volume vital capacity and BMI from two defined time periods (18–26-month period prior to and after Ataluren start). Mean age at loss of ambulation was 10.1 ± 0.5 years, mean age when initiating Ataluren treatment 14.1 ± 1.4 years. Serial echocardiography, pulmonary lung function tests, and assessment of muscle strength indicated mild attenuation of disease progression after initiation of Ataluren treatment. A possible side effect of Ataluren was a reduction in BMI. There were no adverse clinical effects or relevant abnormalities in routine laboratory values. We conclude that Ataluren appears to mildly ameliorate the clinical course in our patients with a good safety profile. However, larger clinical trials are required to assess the role of Ataluren and its long-term impact on disease progression in non-ambulant nmDMD patients.

## Introduction

Duchenne muscular dystrophy (DMD) is an X-linked recessive neuromuscular disease caused by mutations in the dystrophin gene. About 15% of DMD cases are caused by point mutations leading to premature stop codons and disrupted synthesis of the dystrophin protein (1). Stop codon read-through therapy is available with the drug Ataluren (Translarna® by PTC Therapeutics). Following positive results in ambulatory nmDMD (non-sense mutation Duchenne muscular dystrophy) patients (2, 3), Ataluren received conditional approval in ambulant DMD patients aged 5 years and older by the EMA (European Medicines Agency) in 2014. Recently, the EMA recommended approval of expanding the indication of Ataluren to include ambulatory children aged 2–5 years with nmDMD. In 2018 updated guidelines were published by the DMD Care Considerations Working Group (4–6). However, there are limited data on non-ambulatory nmDMD patients treated off-label with Ataluren (4).

Here, we report our experience with Ataluren treatment in four non-ambulatory nmDMD patients.

## Methods

While molecular characterization of patient one and patient two (P1 and P2) is correct, patient 3 and patient 4 (P3 and P4) are siblings sharing the same mutations in exon 51 of the dystrophin gene (c.7513delG; p.Asp2505IlefsX5). While mutations in P1 and P2 predict to lead to a premature stop codon and disrupted synthesis of the dystrophin protein, mutations in P3 and P 4 lead to a frame-shift mutation and a stop-codon. However, this mutation does not qualify for treatment with Ataluren as per manufacturer's specifications. Written informed consent was obtained. Data (echocardiography, spirometry, muscle strength assessment) were collected prospectively every 3–6 months after initiation of Ataluren therapy. All other relevant previous medical data were collected retrospectively (SAP, Walldorf, Germany). Ataluren was administered orally in 3 doses (40 mg/kg/day) as per manufacturer's suggestion and recommendation.

Routine investigations included pulmonary function tests (spirometry), cardiac function (echocardiography) and assessment of muscle strength (Medical Research Council Scale, MRCS). Echocardiographic examinations were performed by the same experienced pediatric cardiologist (HAK). A senior consultant in neurology with extensive expertise in neuromuscular disorders (UD) measured the muscle strength. Routine laboratory values were performed at 6-month intervals.

For comparison of Ataluren associated effects we calculated and compared changes in left ventricular fractional shorting (LVFS) [%], forced volume vital capacity (FVC) [%], FVC [L] and BMI [kg/m^2^] from time period 1 (18–26-month period prior to start of Ataluren to baseline) with time period 2 (18–26-month period after initiation of Ataluren) in all four patients. Baseline was defined as start of Ataluren treatment (Time point 0 in all figures). While systematic assessment of the chosen clinical endpoints the time periods of 18–26 months prior to and after initiation of Ataluren therapy, earlier results from these studies are also depicted graphically.

## Results

### Molecular Characterization

Patient (P) 1 and P2 are identical twins with the same point mutation: c.9100C>T (p.Arg3034Stop) in exon 61 of the dystrophin gene. Of note, after a two-and half year treatment phase with Ataluren, muscle biopsy was performed in P3 and P4, demonstrating subtle dystrophin expression, which was not seen at initial diagnostic biopsies in either patient. Moreover, the clinical course including pulmonary and cardiac dysfunction seems to have been mildly ameliorated during treatment with Ataluren.

### Clinical Characterization

All patients are of European descent and born to non-consanguineous families. All four patients (two families) presented with early developmental delay with prominently delayed motor milestones. All patients achieved independent walking before clinical deterioration. Mean age at loss of ambulation was 10.1 ± 0.5 years; median: 10.3 years, range: 9.5–10.5 years (P1 and P2 10.5 years; P3 10 years; P4 9.5 years).

Systemic corticosteroids were not given to any patient during Ataluren therapy because of treatment associated adverse effects in the past. P1 and P2 had surgical correction for progressive scoliosis 8 months after Ataluren treatment initiation. A progressive scoliosis was noted in P4 2.5 years after Ataluren treatment initiation. All patients received recommended medication in accordance with published guidelines (4–6) (P1: diuretics; P3 and P4: ACE-inhibitors). All patients received supportive care including physical-, occupational- and speech therapy. While the use of supportive respiratory devices (cough assistant, non-invasive ventilation at night) was discussed with and offered to all patients, none of the patients and their family opted for these therapeutic approaches while treated with Ataluren. At the time of initiating Ataluren treatment all four patients were wheelchair-bound.

### Age at Start of Ataluren Treatment

Mean age when initiating Ataluren treatment was 14.1 ± 1.4 years; median: 14.8 years, range: 11.9–14.8 years (P1 and P2 14 10/12 years; P3 14 9/12 years; P4 11 11/12 years). Time interval from age at loss of ambulation until age of Ataluren treatment start was 4.0 years ±1.0 (mean); median: 4.3 years, range: 2.4–4.8 years (P1 and P2 4.3 years; P3 4.8 years; P4 2.4 years). All four patients are currently continuing Ataluren treatment.

### Clinical Investigations

**Figure 1:**
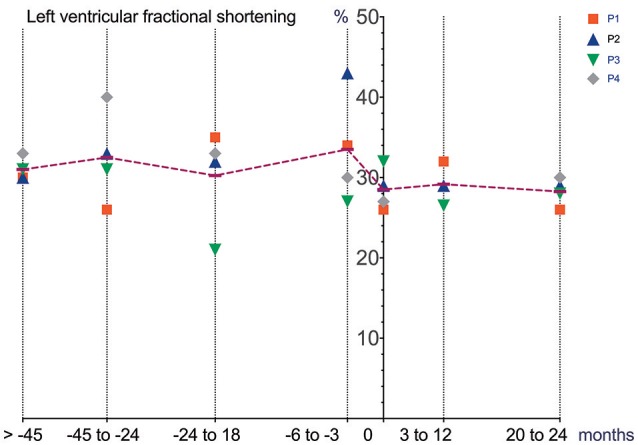
Cardiac function (echocardiography) prior to and after initiation of Ataluren treatment. Graphic representation of LVFS [%] in relation to time. Time point 0 represents start of Ataluren treatment in all figures (P1 and P2 13th October 2015; P3 and P4 5th February 2016). Age at start of Ataluren: P1 and P2 14 10/12 years; P3 14 9/12 years; P4 11 11/12 years). Note that time period's alternate because of a lack of data in some values. LVFS, left ventricular fractional shorting.

**Figure 2:**
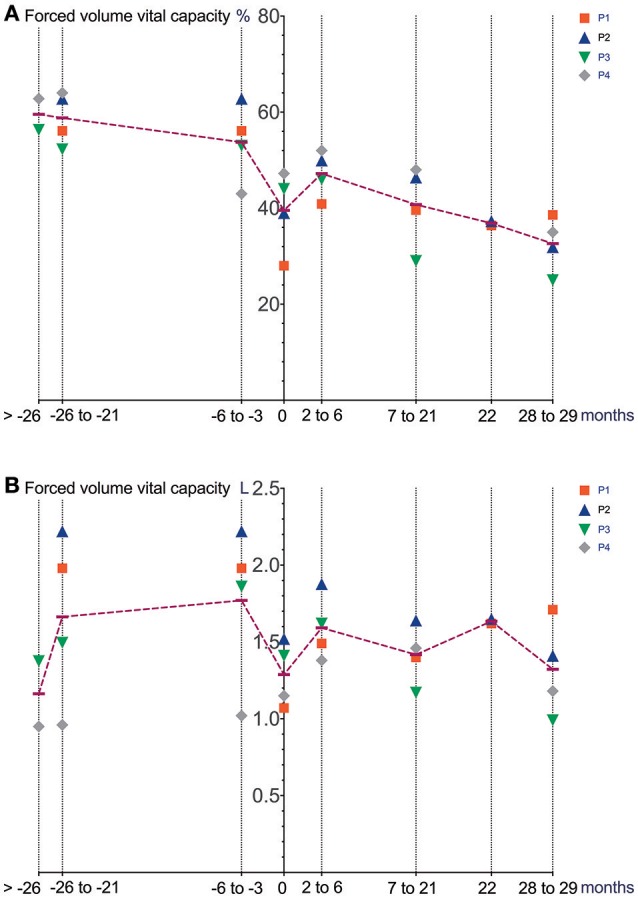
Lung function (spirometry) prior to and after initiation of Ataluren treatment. Graphic representation of FVC [%] **(A)** and FVC [L] **(B)** in relation to time. Time point 0 represents start of Ataluren treatment in all figures (P1 and P2 13th October 2015; P3 and P4 5th February 2016). Age at start of Ataluren: P1 and P2 14 10/12 years; P3 14 9/12 years; P4 11 11/12 years). Note that time period's alternate because of a lack of data in some values. FVC, forced volume vital capacity.

**Figure 3:**
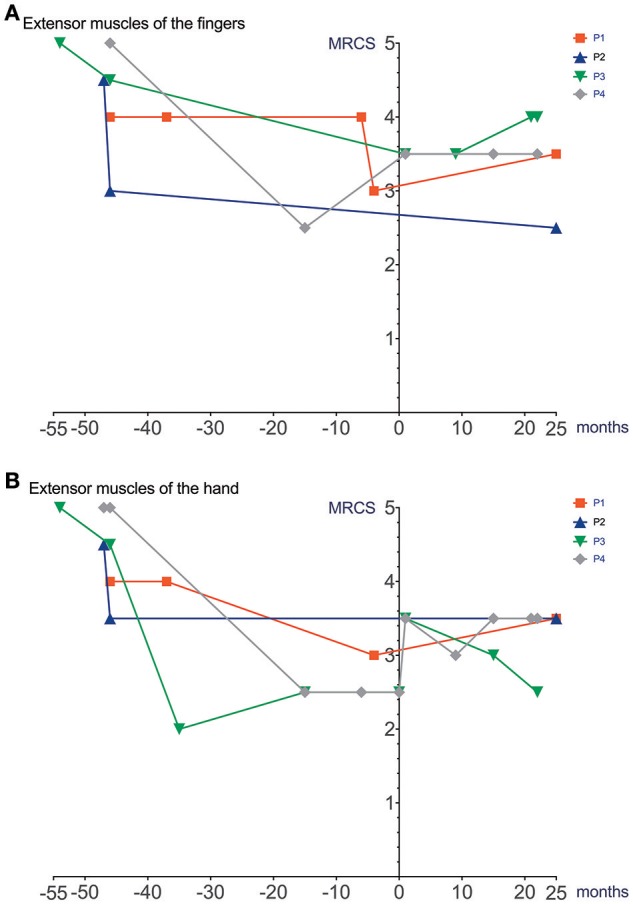
Manual muscle strength prior to and after initiation of Ataluren treatment. Graphic representation of extensor muscles of the fingers (MRCS) **(A)** and extensor muscles of the hand (MRCS) **(B)** in relation to time. Time point 0 represents start of Ataluren treatment in all figures (P1 and P2 13th October 2015; P3 and P4 5th February 2016). Age at start of Ataluren: P1 and P2 14 10/12 years; P3 14 9/12 years; P4 11 11/12 years). Note that time period's alternate because of a lack of data in some values. MRCS, Medical Research Council Scale.

**Figure 4:**
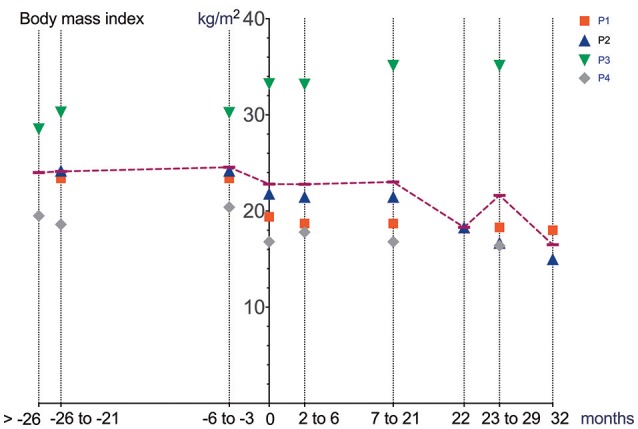
BMI prior to and after initiation of Ataluren treatment. Graphic representation of BMI [kg/m^2^] in relation to time. Time point 0 represents start of Ataluren treatment in all figures (P1 and P2 13th October 2015; P3 and P4 5th February 2016). Age at start of Ataluren: P1 and P2 14 10/12 years; P3 14 9/12 years; P4 11 11/12 years). Note that time period's alternate because of a lack of data in some values.

Cardiac function (echocardiography), lung function (spirometry), manual muscle strength (MRCS) and BMI prior to and after initiation and during treatment with Ataluren are depicted in Figures 1–4.

### Changes in LVFS, FVC, FVC, and BMI

Differences in deterioration of LVFS [%], FVC [%], FVC [L] and BMI [kg/m^2^] between period 1 and 2 are depicted in Table 1. When comparing both time periods, LVFS improved in all patients except for P3. The same was seen in FVC. All changes were not statistically significant.

**Table 1:** Delta changes in LVFS [%], FVC [%], FVC [L] and BMI [kg/m^2^].

		**P1**	**P2**	**P3**	**P4**	**P1-4**
LVFS (%)	Δ Time period 1	−9	−3	+11	−6	−1.75 ± 8.8
	Baseline	26	29	32	27	28.5 ± 2.6
	Δ Time period 2	±0	±0	−4	+3	−0.5 ± 2.9
FVC (%)	Δ Time period 1	−28.1	−23.8	−8.3	−16.8	−19.2 ± 8.7
	Baseline	28	39	44	47.2	39.6 ± 8.4
	Δ Time period 2	+8.4	−1.7	−15	+0.8	−1.9 ± 9.7
FVC (L)	Δ Time period 1	−0.91	−0.7	−0.09	+0.19	−0.38+ 0.51
	Baseline	1.07	1.52	1.41	1.15	1.29 ± 0.21
	Δ Time period 2	+0.55	+0.13	−0.24	+0.31	0.19 ± 0.33
BMI (kg/m^2^)	Δ Time period 1	−4	−1.8	+2.97	−1.95	−1.20 ± 2.95
	Baseline	19.4	21.8	33.2	16.8	22.8 ± 7.23
	Δ Time period 2	−1.1	−3.5	+1.9	−0.4	−0.775 ± 2.22

*Δ Time period 1: changes from 18 to 26 month period prior to start of Ataluren. Baseline: start of Ataluren treatment (Time point 0 in all figures). Δ Time period 2: changes from 18 to 26 month period after initiation of Ataluren. Δ, delta; FVC, forced volume vital capacity; LVFS, left ventricular fractional shorting*.

### Adverse Effects/Routine Laboratory Values

There were no adverse clinical effects related to treatment of Ataluren. No clinically relevant abnormalities in routine laboratory values were seen. A possible side effect related to Ataluren treatment was a reduction in BMI (P2, P3, and P4) and increased BMI in P1 during Ataluren treatment (see Figure 4, Table 1). Subjectively, the patients experienced improved emotional stability as well as better ability to concentrate on tasks. This was also noted by their caregivers and the treating physicians, but not objectively measured.

## Discussion

In this study, echocardiography as well as serial pulmonary lung function and muscle strength assessment possibly demonstrated a mildly attenuated clinical course in four non-ambulatory children and adolescents with DMD treated with Ataluren.

It remains to be elucidated if these medium-term, not statistically significant changes will also translate into longer-term, meaningful clinical benefits. When comparing our data (mean FVC [%] −1.9 ±9.7 over a 18–26-month period after initiation of Ataluren) with data from a natural history cohort study by Mayer et al. (7), it is important to note that the percent predicted FVC showed a near linear decline of approximately 5% points/year from ages 5 to 24 in their cohort. Also, two patients (P1 and P2) underwent corrective surgery for scoliosis 8 months after the start of Ataluren therapy, possibly contributing to a stabilization of pulmonary and cardiac function. Since there was no concomitant corticosteroid use in our patients during Ataluren treatment, the clinical effects seen in our cohort are most likely related to the use of Ataluren. It is important to note that “improvements” in pulmonary function as well as in muscle strength may at least in part be explained by a reduction in BMI, a side effect possibly related to the use of Ataluren. Moreover, the large intraindividual variability makes data interpretation problematic. Other possible confounders (e.g., scoliosis surgery) have to be taken into account. Interestingly, no immediate effect of scoliosis surgery on pulmonary and cardiac function was seen in our patients. Since this report was not a randomized controlled trial, a relevant placebo effect must also be taken into consideration in the assessment of pulmonary function and muscle strength. However, repetitive measurements of left ventricular function are unlikely to be influenced by a placebo effect.

Unfortunately, the subjective improvement in emotional stability and better ability to concentrate on tasks was not assessed using standardized clinical tools (8, 9) because of a lack of baseline data. Since all four patients were non-ambulatory, we used results from routine echocardiography, serial spirometry, and on muscle strength assessment. While this approach encompasses the assessment of important organ dysfunction and is mostly in line with recently published guidelines from the DMD Care Considerations Working Group (5), unfortunately, we did not use more standardized outcome measures for motor function of the upper limb, like the Performance of Upper Limb test (PUL 2.0) or the Brooke Upper Extremity Scale (4, 10, 11). A standardized approach for Ataluren treatment and surveillance in individuals with non-ambulatory nmDMD patients has so far not been published. In a multicentre, randomized, double-blind, placebo-controlled, phase 3 trial in ambulatory nmDMD patients, the primary end point (change in 6MWD from baseline to week 48) was not significant, while *post-hoc* analyses revealed significant differences in a subset of treated boys (i.e., in those with a walking distance of 300–400 m) when compared to non-treated patients (2). Ongoing studies will further investigate these findings in ambulatory boys (e.g., ClinicalTrials.gov identifier: NCT03179631).

Given the rarity of this disease, the number of patients included in our study is limited to a small cohort size, including two brothers and two identical twins. Longitudinal studies with larger sample sizes as well as a registry are needed to further delineate the role and potential of Ataluren to positively impact on the clinical course in non-ambulatory nmDMD patients. Of note, the use of Ataluren demonstrated a good safety profile in our patients.

## Conclusions

To the best of our knowledge this is the first report of off-label use of Ataluren in non-ambulatory nmDMD patients indicating possible clinical efficacy in mildly slowing down the progressive course of DMD disease in this cohort. However, it must be noted that substantial intra- and inter-individual fluctuations were seen in our routine clinical assessment of both pulmonary and cardiac function in our small study cohort. Moreover, other clinical confounders (e.g., corrective surgery for scoliosis) must be taken into account. Thus, further research in larger cohorts is warranted to better delineate the role and potential of Ataluren in non-ambulatory nmDMD patients. Aside from motor function of the upper extremities, cardiac and pulmonary assessment, it will also be important to evaluate cognitive and emotional performance prior to and after the start of treatment with Ataluren. We recommend a consensus conference be held to provide the treating physician with a standardized set of tests for assessment of organ-specific dysfunstion.

## Author contributions

All authors made substantial contributions. DE-F wrote the manuscript to which all co-authors contributed as well, reviewed the clinical case, created all figures and contributed to conceptualization/design, methodology, investigation, data curation and formal analysis and resources. UD treated the patients, performed muscle strength testing, contributed to conceptualization/design, methodology, investigation and critically revised the manuscript for intellectual content. MF-B treated the patients, contributed to conceptualization/design, methodology, investigation and critically revised the manuscript for intellectual content. MP treated the patients and critically revised the manuscript for intellectual content. HA-K treated the patients, performed serial echocardiographies and critically revised the manuscript for intellectual content. MS treated the patients and critically revised the manuscript for intellectual content. BM treated the patients, performed serial pulmonary function tests, contributed to data curation and critically revised the manuscript for intellectual content. MZ contributed to conceptualization/design, methodology and critically revised the manuscript for intellectual content. SM treated the patients, contributed to conceptualization/design, methodology, investigation, supervision/oversight, data curation, formal analysis and resources and critically revised the manuscript for intellectual content. All authors read and approved the final manuscript.

### Conflict of interest statement

The authors declare that the research was conducted in the absence of any commercial or financial relationships that could be construed as a potential conflict of interest.

## Abbreviations

DMD: Duchenne muscular dystrophy
FVC: forced volume vital capacity
LVFS: left ventricular fractional shorting
MRCS: Medical Research Council Scale
nmDMD: nonsense mutation Duchenne muscular dystrophy.
