# Corrigendum: Off-Label Use of Ataluren in Four Non-ambulatory Patients With Duchenne Muscular Dystrophy: Effects on Cardiac and Pulmonary Function and Muscle Strength

**DOI:** 10.3389/fped.2019.00284

**Published:** 2019-07-25

**Authors:** Daniel Ebrahimi-Fakhari, Ulrich Dillmann, Marina Flotats-Bastardas, Martin Poryo, Hashim Abdul-Khaliq, Mohammed Ghiath Shamdeen, Bernhard Mischo, Michael Zemlin, Sascha Meyer

**Affiliations:** ^1^Department of Pediatric Neurology, Saarland University Medical Center, Homburg, Germany; ^2^Department of Neurology, Saarland University Medical Center, Homburg, Germany; ^3^Department of Pediatric Cardiology, Saarland University Medical Center, Homburg, Germany; ^4^Department of Pediatrics, Marienkrankenhaus St. Josef Kohlhof, Neunkirchen, Germany

**Keywords:** Translarna, Duchenne muscular dystrophy, DMD, non-ambulatory, nmDMD, stop codon read-through therapy, pulmonary function test, treatment

Due to incorrect presentation of genetic findings in two of our patients, the article **Title** has been changed from “Off-Label Use of Ataluren in Four Non-ambulatory Patients With Nonsense Mutation Duchenne Muscular Dystrophy: Effects on Cardiac and Pulmonary Function and Muscle Strength” to “Off-Label Use of Ataluren in Four Non-ambulatory Patients With Duchenne Muscular Dystrophy: Effects on Cardiac and Pulmonary Function and Muscle Strength.”

The running title has thus also been changed from “Ataluren in Non-ambulatory nmDMD Patients” to “Ataluren in Non-ambulatory DMD Patients.”

Due to reasons stated above, a correction has been made to the **Abstract:**

“About 15% of Duchenne muscular dystrophy (DMD) cases are caused by point mutations leading to premature stop codons and disrupted synthesis of the dystrophin protein. Stop codon read-through therapy is available with the drug Ataluren (Translarna® by PTC Therapeutics). Following positive results in ambulatory nmDMD (non-sense mutation Duchenne muscular dystrophy) patients, Ataluren received conditional approval in ambulant nmDMD patients by the EMA in 2014. However, there are limited data on non-ambulatory nmDMD patients treated with Ataluren. Here, we report our experience in four non-ambulatory DMD patients. Routine investigations included cardiac function, pulmonary function tests and muscle strength. We compared changes in left ventricular fractional shorting, forced volume vital capacity and BMI from two defined time periods (18–26-month period prior to and after Ataluren start). Mean age at loss of ambulation was 10.1 ± 0.5 years, mean age when initiating Ataluren treatment 14.1 ± 1.4 years. Serial echocardiography, pulmonary lung function tests, and assessment of muscle strength indicated mild attenuation of disease progression after initiation of Ataluren treatment. A possible side effect of Ataluren was a reduction in BMI. There were no adverse clinical effects or relevant abnormalities in routine laboratory values. We conclude that Ataluren appears to mildly ameliorate the clinical course in our patients with a good safety profile. However, larger clinical trials are required to assess the role of Ataluren and its long-term impact on disease progression in non-ambulant nmDMD patients.”

A correction has also been made to the **Discussion** section, paragraph one:

“In this study, echocardiography as well as serial pulmonary lung function and muscle strength assessment possibly demonstrated a mildly attenuated clinical course in four non-ambulatory children and adolescents with DMD treated with Ataluren.”

Additionally, in the original article, there was an error. In the **Methods** and **Results** incorrect information was provided.

A correction has been made to the **Methods** section, paragraph one:

“While molecular characterization of patient one and patient two (P1 and P2) is correct, patient 3 and patient 4 (P3 and P4) are siblings sharing the same mutations in exon 51 of the dystrophin gene (c.7513delG; p.Asp2505IlefsX5). While mutations in P1 and P2 predict to lead to a premature stop codon and disrupted synthesis of the dystrophin protein, mutations in P3 and P 4 lead to a frame-shift mutation and a stop-codon. However, this mutation does not qualify for treatment with Ataluren as per manufacturer's specifications. Written informed consent was obtained. Data (echocardiography, spirometry, muscle strength assessment) were collected prospectively every 3–6 months after initiation of Ataluren therapy. All other relevant previous medical data were collected retrospectively (SAP, Walldorf, Germany). Ataluren was administered orally in 3 doses (40 mg/kg/day) as per manufacturer's suggestion and recommendation.”

A correction has also been made to the **Results**, subsection **Molecular Characterization**:

“Patient (P) 1 and P2 are identical twins with the same point mutation: c.9100C>T (p.Arg3034Stop) in exon 61 of the dystrophin gene. Of note, after a two-and half year treatment phase with Ataluren, muscle biopsy was performed in P3 and P4, demonstrating subtle dystrophin expression, which was not seen at initial diagnostic biopsies in either patient. Moreover, the clinical course including pulmonary and cardiac dysfunction seems to have been mildly ameliorated during treatment with Ataluren.”

The authors apologize for these errors and state that they do not change the scientific conclusions of the article in any way. The original article has been updated.

